# Gastrointestinal problems, mechanisms and possible therapeutic directions in Gulf war illness: a mini review

**DOI:** 10.1186/s40779-021-00341-4

**Published:** 2021-09-09

**Authors:** Diana A. Kimono

**Affiliations:** NYU Langone Medical Center, New York, NY 10016 USA

**Keywords:** Gulf war illness, GWI, Microbiome, Enteric nervous system, Gastrointestinal, GI, Leaky gut

## Abstract

By its nature, Gulf war illness (GWI) is multisymptomatic and affects several organ systems in the body. Along with other symptoms, veterans who suffer from GWI commonly report chronic gastrointestinal issues such as constipation, pain, indigestion, etc. However, until recently, most attention has been focused on neurological disturbances such as cognitive impairments, chronic fatigue, and chronic pain among affected veterans. With such high prevalence of gastrointestinal problems among Gulf war (GW) veterans, it is surprising that there is little research to investigate the mechanisms behind these issues. This review summarizes all the available works on the mechanisms behind gastrointestinal problems in GWI that have been published to date in various databases. Generally, these studies, which were done in rodent models, in vitro and human cohorts propose that an altered microbiome, a reactive enteric nervous system or a leaky gut among other possible mechanisms are the major drivers of gastrointestinal problems reported in GWI. This review aims to draw attention to the gastrointestinal tract as an important player in GWI disease pathology and a potential therapeutic target.

## Background

Gastrointestinal (GI) problems are a commonly reported issue among veterans who return from different wars. These problems developed during or shortly after the wars and persist to date, in a significant number of veterans. The most common GI issues reported include diarrhea, dyspepsia, heartburn, noncardiac chest pain, functional gastrointestinal disorders, constipation, etc. and they have been summarized in several reviews [1, 2]. The exact causes of these issues are difficult to pinpoint, but Wang et al. [1] argues that they developed most likely due to unsanitary conditions, the diet, stress, psychological issues and some chemical exposures before the war or in the war theatre.

Among veterans who returned from the Persian Gulf war (GW) of 1990–1991, GI problems are highly prevalent and occur concurrently with several of the other symptoms that the veterans present [2, 3]. Interestingly, although GI issues are common among veterans from different wars, they seem to occur at a higher frequency among GW veterans and they continue to persist many years after the war [4]. Literature does not agree on the exact numbers of GW veterans who are affected by GI problems. However, from different cohort studies, between 14–25% of GW veterans report from GI disturbances [3, 5–8].

With this high prevalence of GI problems among GW veterans, it is surprising that little attention has been focused on understanding the nature and mechanisms driving these issues. So far, most attention has been focused on neurological disease mechanisms in Gulf war illness (GWI) [9–14]. However, the past decade has seen an emerging interest in scientific studies which investigate the physiological and molecular mechanisms underlying GI problems in GW illness. Most of these studies are now focusing on a gut-brain axis as opposed to merely looking at the brain alone in order to explain the pathology of these conditions [15–17].

This review summarizes the only studies that have been published to date, which provide detailed mechanistic pointers to explain GI problems observed among GW veterans. In summary, these studies propose three mechanisms to explain the source of inflammation observed in GWI sufferers. They suggest that an altered microbiome, compromised gut integrity and a dysfunctional enteric nervous system which resulted from chemical exposures and war stress are the major drivers of the persistent GI inflammation observed in GWI. The studies were done using rodent models, in vitro assays and by studying various human GW veteran cohorts.

## An altered microbiome as a source of immunogenic particles

The gut microbiome is a population of bacteria, viruses, fungi, protozoans, etc. that exist naturally along the gut, from the mouth to the anus [18–20]. The role of these organisms has only recently been appreciated as critical to the optimal functioning and wellbeing of their host [21]. In several diseases and disorders such as autism, Parkinson’s, Alzheimer’s, diabetes, irritable bowel syndrome, etc. an altered microbiome has been found to be an important contributor to their pathology. In Crohn’s disease, for example, Henke et al. found and characterized an inflammatory polysaccharide which is produced by the gut bacteria *Rumminococcus gnavus,* which have been observed to proliferate during flares of patients with Crohn’s disease. This polysaccharide is known to induce the production of tumor necrosis factor-α (TNF-α), which contributes to the inflammation observed in Crohn’s disease [22]. Also, in diabetes it has been consistently found that there is a decrease in the relative abundance of Firmicutes and Clostridia and an increased abundance of Bacteriodetes. This altered Firmicutes: Bacteriodetes ratio correlates with blood glucose levels in diabetic patients [23, 24]. In Parkinson’s disease (PD), Baldini et al. [25] found a significant change in 8–9 bacterial genera of PD patients compared to the controls and a correlated increase in certain bacterial metabolites known to contribute to the development of PD. With such observations, there is an increasing focus on the microbiome as a therapeutic target in several disorders [26–29]. Potential approaches target the microbiome by attempting to restore it to normal or near normal levels. This can be done through providing substrates that support restoration of probiotic bacteria (e.g. butyrate producing bacteria) such as a diet rich in fiber [30] or supplementation with short chain fatty acids, for example in the study by Seth et al. [16]. It can also be achieved through replacing the microbiome via fecal transplants [31]. Both these approaches have registered promising success in the treatment of the several disorders.

### An altered bacteriome in GWI rodent models

In the study by Alhasson and colleagues, exposure to GW chemicals was associated with a significant alteration of the bacteriome of mice treated with pesticides and prophylactic drugs (to represent GW chemical exposures in the war theatre). Their study aimed to examine the effects of GW chemical exposure on the bacteriome as a key pathway to neuro- and gastrointestinal inflammation via systemic endotoxemia and toll-like receptor pathway activation. They used a murine model of GW illness in which mice were treated with permethrin (200 mg/kg) and pyridostigmine bromide (2 mg/kg) three times a week, for one week. This was followed by a daily dose of corticosterone applied intraperitoneally (100 μg/mice/day) for 5 days. To control for the effect of the gut bacteriome, they attempted to decontaminate the gut through the use of broad-spectrum antibiotics, neomycin (45 mg/kg) and enrofloxacin (1 mg/kg) thrice a week for two weeks [15].

Their studies found that GW chemical exposure resulted in gut dysbiosis at both phylum and family levels of normal bacteria flora. GW chemical treated mice had significantly increased abundance of Firmicutes and Tenericutes over Bacteriodetes as compared to the vehicle control treated mice. Further, there was a significant increase in Ruminococcaceae and other two unclassified families in GW treated mice. They also reported significant differences in beta diversity of bacteria in the gut lumen of mice exposed to GW chemicals, and a significant increase in Shannon diversity of bacteria and 109 significantly different bacterial operational taxonomic units (OTUs) in these mice compared to controls.

Associated with the changes observed in the bacteriome, this study also found a loss in gut barrier integrity. They saw a decrease in tight junction proteins claudin 2 and increase in occludin expression in the small intestine, which correlated with increased serum endotoxin levels in mice treated with GW chemicals compared to controls. And finally, they reported an increase in toll-like receptor 4 (TLR4) mediated oxidative stress and inflammation in GW chemical treated mice.

Their results generally propose that exposure of GW troops to pesticides and prophylactic drugs such as pyridostigmine bromide coupled with war stress could have resulted in an alteration of their microbiome. This caused inflammation and systemic endotoxemia due to a leaky gut. These endotoxins in the blood then reach peripheral organs such as brain, liver, etc. contributing to the multisymptomatic nature of the disease.

Further, the same group provided evidence that these microbiome alterations actually persist long after GW chemical exposures were stopped [32]. The study by Kimono et al. [32] reported that microbiome alterations and increased levels of immunostimulatory particles such as high mobility group box 1 (HMGB1) and low systemic endotoxemia persisted in mice treated with GW chemicals compared to those treated with vehicle controls 20 weeks after GW chemical exposures were stopped. Although the study did not conclusively prove that the altered microbiome was the major source of these particles, they performed statistical correlation analyses that showed a strong correlation between these immunostimulatory particles and the microbiome. However, it would be valuable to investigate this issue further.

Following these studies, other important questions arise. For example, can other factors known to affect microbiome composition worsen GI and other symptoms of GWI? In a study by Angoa-Pérez et al. [33], it was reported that the taxonomic structure of the gut microbiome was not only altered by exposure to GW chemicals but that these changes became more significant in mice which were fed on a high fat diet. This study was motivated by reports that a growing number of GW veterans are obese [34]. The work also found that when the mice were returned to a normal chow diet (to represent a healthy diet), the gut microbiome structure was restored to near normal levels [33].

Further, another study by Bose et al. [35] supports the findings that indeed, a high fat diet worsens alterations to the microbiome in GW chemical exposed mice, and inflammatory GI pathology. Their study demonstrated that when mice which had been exposed to GW chemicals [permethrin and pyridostigmine bromide (PB)] for 2 weeks (to represent the war phase) were fed on a Western diet for 5 months after the exposure to GW chemicals (to represent post war phase), there was a significant decrease in butyrogenic and other health preserving bacteria compared to mice which had been fed on a normal chow diet. This shift in the microbiome composition toward an “unhealthy” microbiome profile was accompanied by increased serum proinflammatory cytokine interleukin-6 (IL-6), and inflammation in the distal small intestine marked by high Claudin 2, IL-6 and interleukin-1β (IL-1β) levels. The authors highlight that these changes in microbiome and increase in inflammation are worse in the obese mice compared to lean mice [35].

In all these studies, the authors acknowledge that their work had some limitations, for example, they used mice whose gut had been decontaminated with broad-spectrum antibiotics as a control for the effect of the gut microbiome. However, it would have been more ideal to use germ-free mice. That notwithstanding, their work has provided deeper insights into understanding the pathology of GW illness and has resulted into further research on the gut microbiome in GW illness [36–39] and other war related diseases.

### An altered virome in GWI mouse models

Most studies concerning the microbiome focus mostly on the bacteriome. However, the microbiome is not composed of only bacteria but several other microorganisms including fungi, protozoans, and viruses. Viruses and their interactions with other microorganisms like bacteria have been reported to impact health [40]. Zuo et al. [41] reported an altered rectal mucosal virome in patients with ulcerative colitis and this correlated with intestinal inflammation while Moazhen and colleagues [42] discovered a link between the gut virome and hypertenstion, and this virome signature was distinct between healthy, pre-hypertensive and hypertensive patients.

In GWI, Seth et al*.* [43] used an established mouse model to study the effect of pesticides and pyridostigmine bromide exposure on the interactions between the gut virome and bacteriome and associated inflammation. They hypothesized that the use of antibiotics and antivirals restores the microbiome community to a healthy state and ameliorates inflammation in GWI. Their experimental model consisted of 4 groups of mice (*n* = 11); vehicle control, GWI (permethrin 200 mg/kg), PB (2 mg/kg), GWI + AB (permethrin + PB and antibiotics-enroflaxacin 1 mg/kg and neomycin 45 mg/kg), GWI (permethrin + PB and broad spectrum antiviral rivabarin 100 mg/kg). Virome and bacteriome population dynamics were studied from mouse stools. They found that indeed there was a significantly altered gut virome in GWI mice compared to controls. Exposure to GW chemicals resulted in an increase in virome richness and relative abundance of double strand DNA bacteriophages including Myoviridae, Siphoviridae and Caudovirales but a decrease in the relative abundance of the single strand DNA bacteriophages Microviridae. When rivabarin was applied, they found that the enteric viral community was restored to near the control group. They further suggest that the interactions between the bacteriophages and their host bacteria significantly drive the observed inflammatory and neurotoxicity observed in GWI. Their results show that treatment with rivabarin improves gastrointestinal barrier integrity loss in GW chemical treated mice, intestinal and systemic as well as neuroinflammation. They also showed that these interactions between bacteria and viruses activate the innate immune system through triggering toll-like receptors 7 and 9 (TLR7 and TLR9) proinflammatory pathways, and a positive correlation between virome diversity and serum IL-6 levels in GW chemical treated mice.

However, the authors point out that their study is imperfect due to the lack of a more robust GWI model since pesticides were applied orally rather than the dermal route and the lack of germ-free mice to control for microbiome and that their model only accounts for the acute phase of GWI. That aside, their study is significant because it shines a light in a previously unexplored area in GWI, proposing that bacteriophages are an important player in driving altered microbiome associated inflammation and immunotoxicity in GWI. They further suggest the use of antivirals as possible therapies for GWI.

### Altered microbiomes in GW veteran cohorts

In a study by Janulewicz et al. [44], a small cohort of veterans from the Boston consortium was studied for changes in their microbiome and inflammation. This study reports on an altered bacteriome in GW veterans who suffer from GWI and report GI disturbances. It was carried out on a subset of veterans from the Boston Gulf war illness consortium, who agreed to participate in a pilot call back study and provide a stool sample. In total, their sample size was 30 veterans, with 7 controls (did not go to the GW), 5 veterans who have GWI but no gastrointestinal problems (GWI-GI) and 14 GW veterans with GWI and GI problems (GWI + GI). Participants filled a questionnaire, provided a stool sample, and a blood sample too. The stool samples were processed, and bacteriome population dynamics were analyzed for all groups, while the circulating proinflammatory cytokines were analyzed from blood serum.

The results of this study reported that there was a significantly altered microbiome in veterans who present with GWI and GI issues compared to controls. And even more interesting, they report that there is also a significant difference between veterans with GWI and GI issues, and those with GWI but no GI issues. Their work found that there was a significantly elevated level of the soluble receptor for tumor necrosis factor-receptor 1 (TNF-R1) in subjects who reported GWI with GI problems compared to controls or those who do not have any GI symptoms (*P* < 0.05). Further, they found that there was a significantly lower bacterial species richness as well as diversity in controls compared to GW veterans with GI issues. The GWI + GI group had a greater abundance of the phyla Bacteroidetes, Actinobacteria, Euryarchaeota, and Proteobacteria as well as higher abundance of the families Bacteroidaceae, Erysipelotrichaceae, and Bifidobacteriaceae when compared to the groups of GW-control veterans and GWI-GI veterans.

This study is important because it supports the studies which have been performed in animal models and paves way for further studies on larger cohorts, and development of microbiome targeted therapies in GWI. However, the study is limited mainly because of its small sample size, although the authors indicate that larger cohort studies are underway.

## A Leaky gut as a portal for immunostimulatory particles

Several gastrointestinal conditions are characterized by intestinal permeability or leaky gut syndrome [45–48]. In this condition, inflammation results in the loss of gut barrier integrity and hence a leakage of intestinal luminal contents into the portal circulation resulting in an inflammatory response with in the GI tract and in peripheral organs [49]. In diabetes, for example, the study by Thaiss et al. [50] reports that hyperglycemia drives gut permeability through GLUT-2 alteration of epithelial cells barrier functions. In their prospective study, Chang et al*.* [51] found that patients who had symptomatic inflammatory bowel disease (IBD) also presented with a leaky gut. While in amylotrophic lateral sclerosis (ALS), Wu et al. [52] reported that a damaged tight junction structure i.e., zonula occludens-1 (ZO-1), adherens, and E-cadherin protein structure and an increased intestinal permeability were observed in their ALS mouse model.

Among GW veterans, the study by Zhang et al. [8] reports intestinal hyperpermeability in veterans who report GI symptoms. The study worked with veterans from The Cincinnati Veterans Affairs Medical Center (VAMC) in Cincinnati, OH and the Malcom Randall VAMC in Gainesville, FL. Veterans underwent a 7-day period of monitoring their abdominal and bowel movement habits. After the 7th day, they had a 24-h urine sample collection following consumption of a mannitol/lactulose solution. Mannitol and lactulose concentrations were then measured in the urine samples. This study was carried out in a cohort of 73 male GW veterans and measured GI permeability by use of the urine lactulose/mannitol test. They then correlated the results of these tests with abdominal pain ratings, frequency of bowel movements and consistency of the bowel movements using the Bristol Stool scale. They found that 39% of the veterans had a significantly higher intestinal permeability, higher daily stool frequency, increased liquid stools, and a higher abdominal pain rating. The study further found a significant correlation between intestinal permeability and abdominal pain among GW veterans who reported GWI.

This study provides support to a proposed mechanism of a leaky gut, which had only been shown in rodent models [15, 16]. Further, this study is important because it provides evidence in humans of a leaky gut syndrome in GWI, and therefore intestinal barrier loss can be considered as a therapeutic target for GWI-related GI disturbances. However, their study suffers some limitations such as recall bias, since it was carried out over 20 years after the war. Also, most of the subjects who took part in the study were Gulf War Veterans enrolled in only two GW registries i.e. the Gulf War Registries at the Malcom Randall VAMC and at the Cincinnati VAMC only. It is therefore not clear if these findings can be applied to all veterans who returned from the Gulf war, and therefore further studies are needed to support this study. Finally, it would also be interesting to see if the same results apply to female veterans of the GW, since this study only included male subjects.

## Enteric nervous system dysfunction as a potentiator of inflammation

Neurological problems have been widely reported among veterans who suffer from GWI [2, 53, 54]. Studies into these issues have focused largely on the central nervous system (CNS) and report several observable and mechanistic findings [9, 12, 34, 55]. To date, very few studies have looked at the effect of GW chemical exposures on the enteric nervous system (ENS). This is surprising considering the number of veterans who report GI issues together with neurological issues. Moreover, it is often found that in neurological disorders, patients often report debilitating GI disturbances which significantly add to a poor quality of life [56]. Since the enteric nervous system shares a lot of similarities with the central nervous system, it is not surprising that in neurodegenerative disorders such as Alzheimer’s [57] and PD [4, 28, 58] studies report similar neurodegenerative pathology in the ENS nerves as in the CNS of affected patients. This explains the decline in GI functions which presents symptoms such as constipation, dysphagia, diarrhea, pain, blotting, etc. reported in patients with neurodegenerative disease [15, 59, 60].

In GW illness, sufferers generally report both neurological and several GI issues including pain, poor GI motility, etc. although there have been few possible mechanisms to explain these symptoms. So far, there were only three studies that report on possible enteric nervous system dysfunction as a major contributor to GWI pathology.

The study by Zhou et al. [61] reports somatic hypersensitivity in veterans with GWI and with GI symptoms. This study recruited previously deployed GW veterans who reported GI symptoms (*n* = 53), veteran controls (*n* = 38) and veterans who had GWI but not GI issues. In this study, veterans with GWI and gastrointestinal symptoms reported a lower pain threshold for the different stimuli tested. Although they do not directly study the enteric nervous system, the fact that GWI sufferers have a disconnect in the perception of pain, which is transmitted along the different nerves GI nerves to the brain and spinal cord, is a strong pointer to a dysfunctional enteric nervous system.

In another study, Hernandez et al. [17] used a murine model of GWI to study the effect of PB on GWI. PB is a reversible acetylcholinesterase inhibitor prophylactic drug which was administered to GW veterans while in the war theatre to protect them from potential exposure to nerve agent sarin [62, 63]. Although not all veterans consumed the drug, about 250,000 personnel reported having used it at least once during deployment, since consumption of the drug was to be as needed or only when ordered.

The authors tested the hypothesis that PB alters gut function by disrupting the neural and immune systems of the intestine. They exposed both male and female mice to two doses of PB (9 and 90 mg/L respectively) through drinking water. The mice were then euthanized after 7 or 30 days to represent short-term and long-term effects of PB exposure on GW veterans, respectively. They found that PB drove acute alterations in colonic motility and fecal fluid content in both male and female mice, in a dose dependent manner. And, the acute exposure to PB was enough to cause persistent changes in colonic motility.

They also observed that PB exposure was associated with significant persistent changes in neurogenic contractions in male mice, and no significant effect on neurogenic relaxations. In female mice, they found that that PB exposure was associated with divergent effects and neurogenic contractions in the acute model while it increased neurogenic contractions in their persistence model. These results were suggestive of altered enteric neuron functioning. The in vivo and ex vivo data from their study indicate that PB has lasting effects on colonic contractility and motility that involve differing mechanisms in female and male mice. They further looked at the effects of PB on enteric neurons and glia. They found that PB initially increases the activity of enteric neurons and glia but that its continued presence in the gut leads to an overall decrease in sensitization of cellular signaling with in the ENS. They also found that PB exposure was associated with both acute and persistent glial reactivity with increased production of nitric oxide. The mice also showed signs of significant neuronal degeneration as in the acute model. Interestingly, they found that female mice did not exhibit changes to glial reactivity or neuronal density following PB exposure, but instead exhibited a shift in the proportion of excitatory neurons. They found that PB exposure mainly resulted in a general decrease in proinflammatory mediators in the colon, and this effect is more pronounced in male mice. In the brain, they found that exposure to PB resulted in disruption to the proinflammatory cytokines and chemokines of male mice, and that in female mice these changes occur more slowly.

This study is important because it shows that at least one chemical exposure in GW illness could account for the development of neuroinflammation in the enteric nervous system with enteric gliosis and neurodegeneration. It is also an important study because it reports that these effects are sex dependent. These findings could lead to advancements in developing novel therapies for GI issues in GWI.

In another study, Kimono et al. [64] report that redox imbalances in enteric glia modulate changes in tight junction protein expression. In this study, the authors used a murine model of GWI in which they exposed the mice to PB (2 mg/kg) and permethrin (200 mg/kg) and corticosterone (100 µg/kg), and another group of mice with broad spectrum antibiotics (neomycin; 45 mg/kg and enrofloxacin; 1 mg/kg) to control for the effects of the microbiome. In this study, they hypothesized that exposure to GW chemicals alters the microbiome resulting in an increased stool endotoxin levels and damage associated molecular patterns (DAMPS) such as HMGB1 which in turn results in glial reactivity and redox imbalances through activation of toll like receptors and nicotinamide adenine dinucleotide phosphate (NADPH) oxidase 2 (NOX2) with production of nitric oxide and inflammation. This continued glial reactivity then modulates the expression of tight junction proteins; claudin 2, occludin, ZO-1 and claudin 1 expression. This study is significant because it raises several important considerations for further investigation. For example, the authors propose the use of TLR4 blockers like Sparstolonin B (SsnB) and short chain fatty acids such as butyrate as potential therapies to target reactive glia in GWI pathology.

## Conclusion

The last decade has seen great strides towards understanding the mechanisms behind GWI pathology. This in part is due to the condition finally being acknowledged as a medical issue and therefore opening the way for further research and funding towards advancing our knowledge, and to develop possible therapies in the next few years. However, even with these developments, most research effort is still largely focused on studying central nervous system disorders in GW veterans and only a few studies currently report on GI issues. These studies generally show evidence that GI issues in GWI are associated with an altered microbiome, gastrointestinal permeability with increased circulating endotoxin levels and finally enteric nervous system dysfunction.

An altered gut microbiome which is characterized by an imbalance in bacterial populations results in the increase in pathogen associated molecular patterns e.g., bacterial parts which through the activation of toll like receptors causes oxidative stress and inflammation. This proinflammatory environment in the gut triggers mechanisms that lead to the loss of tight junction integrity between epithelial cells, and poorly functioning water channels. This results in a loss of gut barrier permeability and a leaky gut, where luminal particles which usually do not enter the blood circulation can pass and reach other peripheral organs. The particles may fuel a chronic inflammatory response in other organs such as in the brain and the liver [15, 16, 35, 43]. Evidence of a chronic inflammatory response has been reported in studies on GW veteran cohorts, where blood samples are taken and analyzed for biomarkers of inflammation. These studies have reported dysregulation of immune cells and other inflammatory markers such as C-reactive protein, Tau proteins, complement proteins, cytokines and chemokines [13, 65–67]. This may in part explain why patients with GWI report multiorgan disorders whose causes are difficult to pinpoint. Gut dysbiosis may also result in enteric nervous system dysfunction through redox imbalances in enteric nervous system, although this still needs to be investigated further. It is also noteworthy that GW chemicals could have been directly toxic to the ENS resulting in chronic inflammation and poor colonic motility as suggested by the study by Hernandez et al. [17]. With these mechanisms coming to light there is hope for specifically targeting the identified pathways to relieve or reverse the symptoms of GWI.

## Data Availability

Not applicable.

## Abbreviations

ALS: Amylotrophic lateral sclerosis
CNS: Central nervous system
DAMPS: Damage associated molecular patterns
ENS: Enteric nervous system
GI: Gastrointestinal
GW: Gulf war
GWI: Gulf war illness
HMGB1: High mobility globulin box group 1
IBD: Inflammatory bowel disease
IL-1β: Interleukin-1β
IL-6: Interleukin-6
NADPH: Nicotinamide adenine dinucleotide phosphate
NOX 2: NADPH oxidase 2
OTUs: Operational taxonomic units
PB: Pyridostigmine bromide
PD: Parkinson’s disease
SsnB: Sparstolonin B
TLR4: Toll-like receptor 4
TLR7: Toll-like receptor 7
TLR9: Toll-like receptor 9
TNF-α: Tumor necrosis factor-α
TNF-R1: Tumor necrosis factor-receptor 1
VAMC: Veterans Affairs Medical Center
ZO-1: Zonula occludens-1
